# Timely Diagnosis of Basilar Artery Occlusion Leading to Improved Outcomes: A Case Report

**DOI:** 10.7759/cureus.41096

**Published:** 2023-06-28

**Authors:** Jonathan E Hanson, Syed Hashim Ali Inam, Lindsay Littlehales, Jason Adams, Paul H Blom, Justin Nolte

**Affiliations:** 1 Neurology, Marshall University Joan C. Edwards School of Medicine, Huntington, USA; 2 Interventional Radiology, Cabell Huntington Hospital, Huntington, USA

**Keywords:** stroke treatment, stroke protocol, marshall university, inam, hanson, posterior circulation stroke, posterior circulation intervention, basilar artery occlusion, basilar artery

## Abstract

This is a case report of an 83-year-old female who presented to the emergency department within eight hours of symptom onset. A CT angiogram revealed a distal basilar artery occlusion, as well as a perfusion deficit in the right superior cerebellar artery. Her symptoms fluctuated, and she was started on a heparin drip, but later in the evening her symptoms worsened. A mechanical thrombectomy was performed by interventional radiology. The following morning, most of the patient’s deficits had resolved, and, when seen in the clinic several weeks later, she continued to be asymptomatic. This case report highlights the importance of timely diagnosis and intervention in the management of distal basilar artery occlusion.

## Introduction

Twenty percent of all ischemic strokes are attributed to posterior circulation. Although only 1% of all ischemic strokes are caused by basilar artery occlusion (BAO), they are potentially the most devastating, with mortality from BAO without recanalization being 75%-96% [1].

BAO can present with a spectrum of symptoms, ranging from mild, transient ones to severe life-threatening strokes with significant morbidity and mortality. BAO can also present with non-specific symptoms such as vertigo, headache, altered mentation, numbness or tingling, and speech or swallowing issues, which sometimes makes it challenging to diagnose. Early recognition and treatment are crucial to the good prognosis of these patients. Intravenous or endovascular intervention is the mainstay of BAO treatment, which restores blood flow in the occluded artery and rescues the brain tissue [2].

The literature review showed that the recanalization of acute BAO is associated with reduced mortality (by half) as well as decreased risk of death or dependency (by 1.5 times) [3]. Case reports have suggested that patients with BAO can benefit from interventional procedures beyond eight hours after symptom onset [4]. Another study, though underpowered, showed the benefit of endovascular therapy in acute BAO [5]. Therefore, early recanalization therapy is associated with better outcomes in BAO patients [6].

We report a patient who presented sudden-onset facial and left-sided numbness, dry mouth, nausea, and mild dysarthria. This case report emphasizes how even in the absence of cerebellar or brainstem signs, magnetic resonance imaging (MRI) and magnetic resonance angiography (MRA) should be considered, as they can aid in the timely diagnosis of life-threatening vertebrobasilar artery occlusion.

## Case presentation

Our patient was an 83-year-old woman with a past medical history of atrial fibrillation (AF), hypothyroidism, cataracts, and sleep apnea who presented to our facility as a stroke alert with complaints of nausea, dry mouth, and tingling on the left side of the body. The patient reported being nauseous the previous night before going to bed but went to sleep thinking she may have been sick from her dinner. At around 04:30 she woke up with difficulty speaking due to a dry mouth, tingling on the left side of her body, and mild dysarthria. Emergency medical services (EMS) was called, and the patient was brought to the hospital. Notably, the patient had stopped her home rivaroxaban the morning before due to a planned cataract surgery later in the week. Her other home medications included levothyroxine, calcium supplements and multivitamin pills that were continued during hospitalisation.

**Figure 1:**
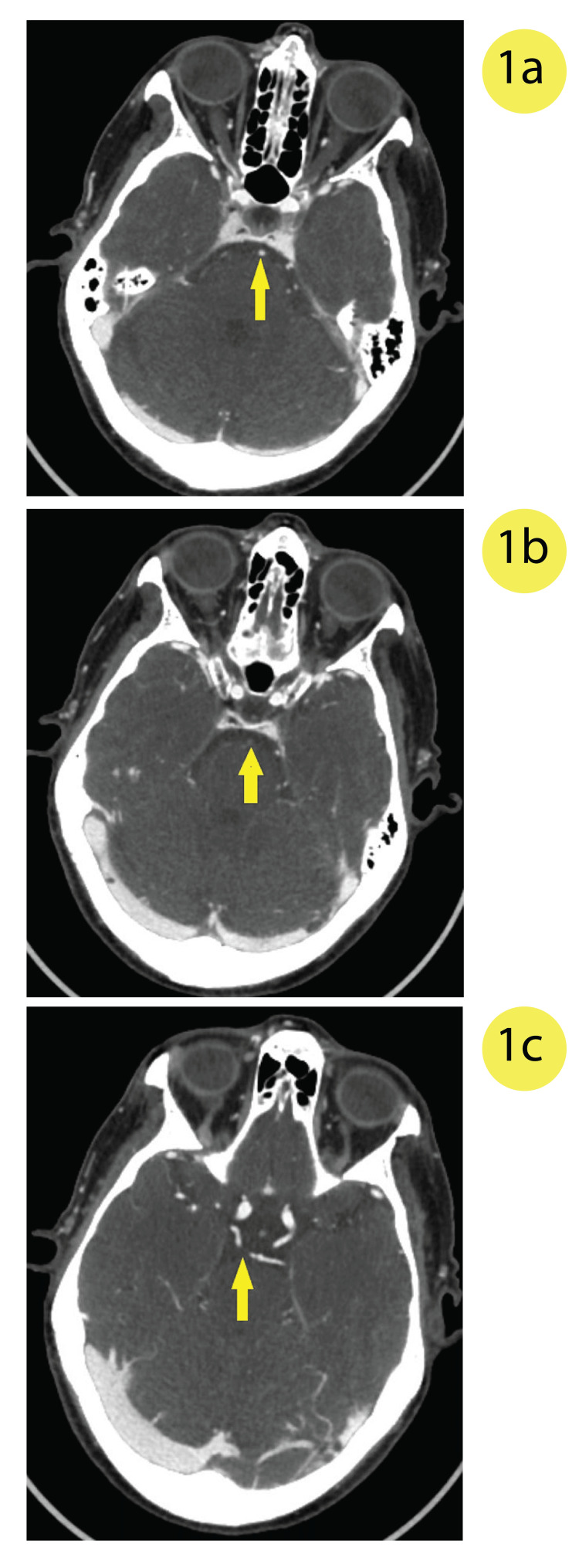
CT angiogram of the head showing a) the basilar artery just inferior to the occlusion, b) the basilar artery at the level of the occlusion, and c) reduced flow in the right superior cerebellar artery

**Figure 2:**
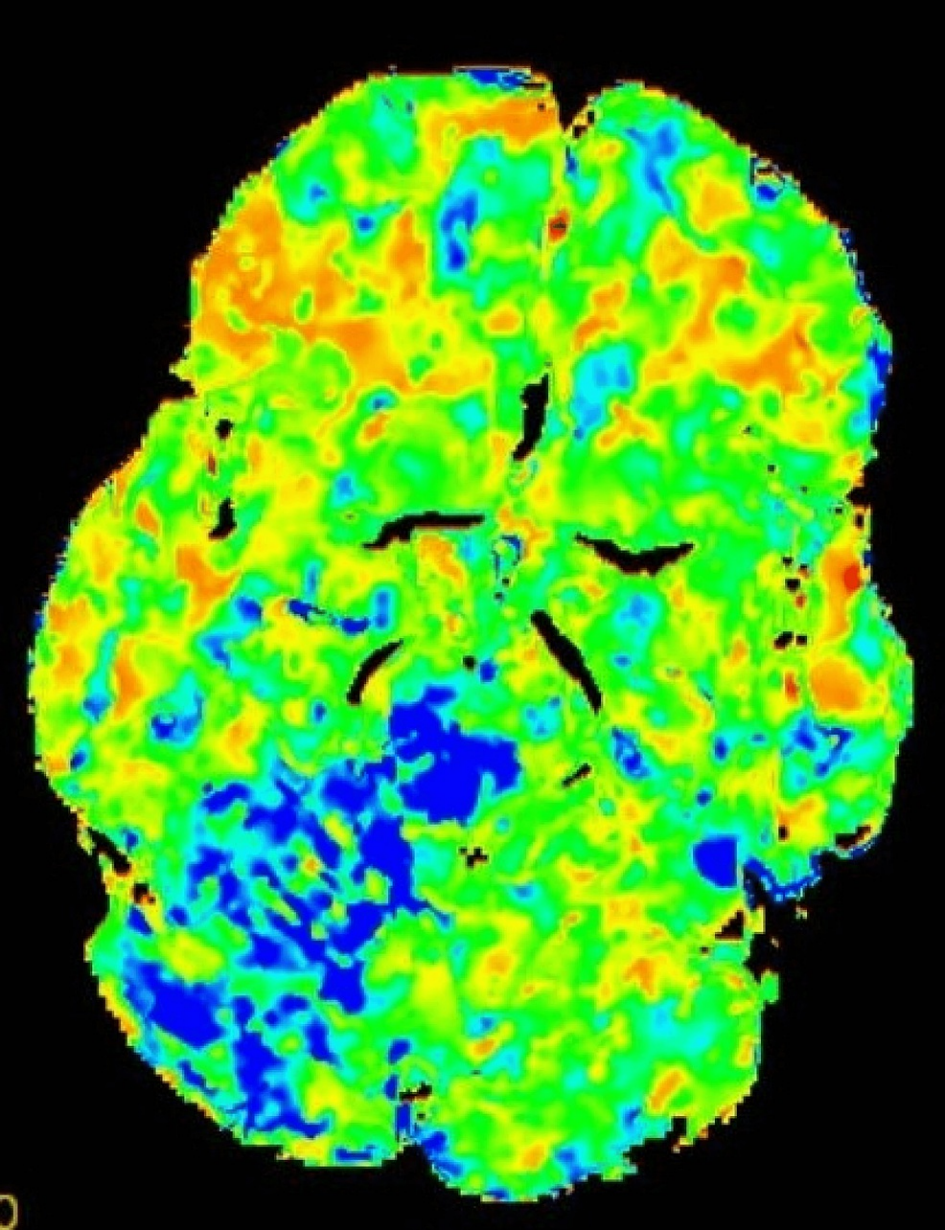
Perfusion imaging showing increased mean transit time (MTT) in the right superior cerebellar artery territory

The patient came to our facility via EMS as a stroke alert around 06:00. Her last known normal (LKN) was determined to be 22:30 the previous night. On arrival, her blood pressure was 150/80, her blood glucose level was 101 mg/dL, her heart rate was in the 70s (beats per minute), and there was active AF with a right bundle branch block on electrocardiogram. She reported no pain she was afebrile. National Institutes of Health Stroke Scale (NIHSS) was 1 for mild dysarthria; no motor deficits or weakness was appreciated on her initial examination. Glasgow Coma Scale (GCS) was 15, fully alert and oriented. Her pupils were equal and reactive on both sides. Modified Rankin Scale (mRS) score was 1. Echocardiogram reveal an ejection fraction of 55-60%, bubble study was negative and no shunt was noted with contrast study. On basic labs, her white blood cell count was 5.55 k/cmm, hemoglobin 11 gm/dL and platelets were 240 k/cmm. Potassium was 4.5 mEq/L, sodium levels were 135 mEq/L, creatinine was 0.91 mg/dL. Other basic labs were unremarkable. Urine analysis was negative. Patient denied diarrhea or constipation. Patient reported mild nausea which she thought was due to the lunch she had that day. The immediate CT of the head was reported as normal. A CT angiogram of the head and neck revealed an occlusion of the distal basilar artery with a cerebral perfusion deficit of the right superior cerebellar artery (Figures 1, 2).

**Figure 3:**
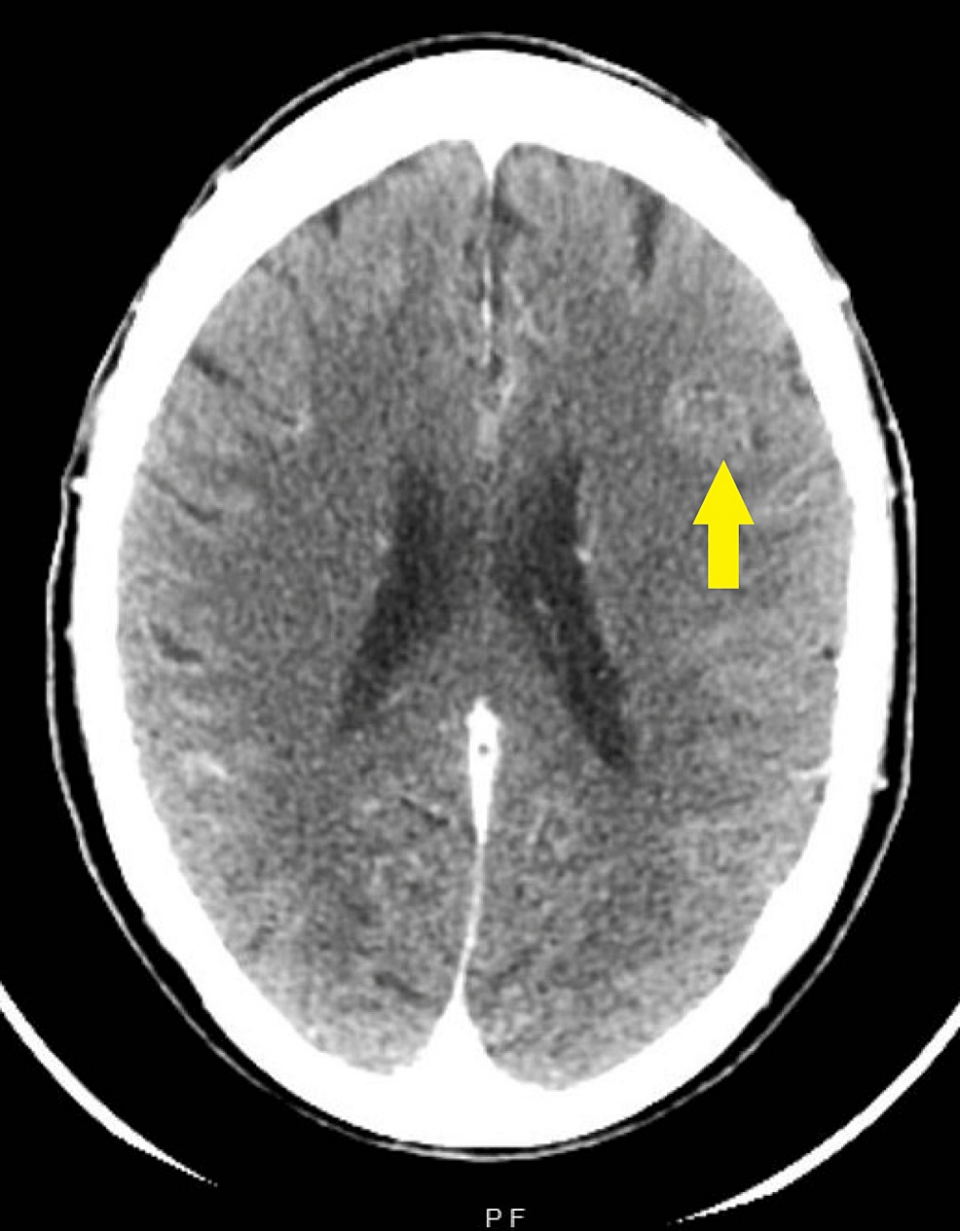
Post-contrast head CT. Arrow is pointing to the questionable left frontal lobe lesion that seemed to enhance with contrast.

**Figure 4:**
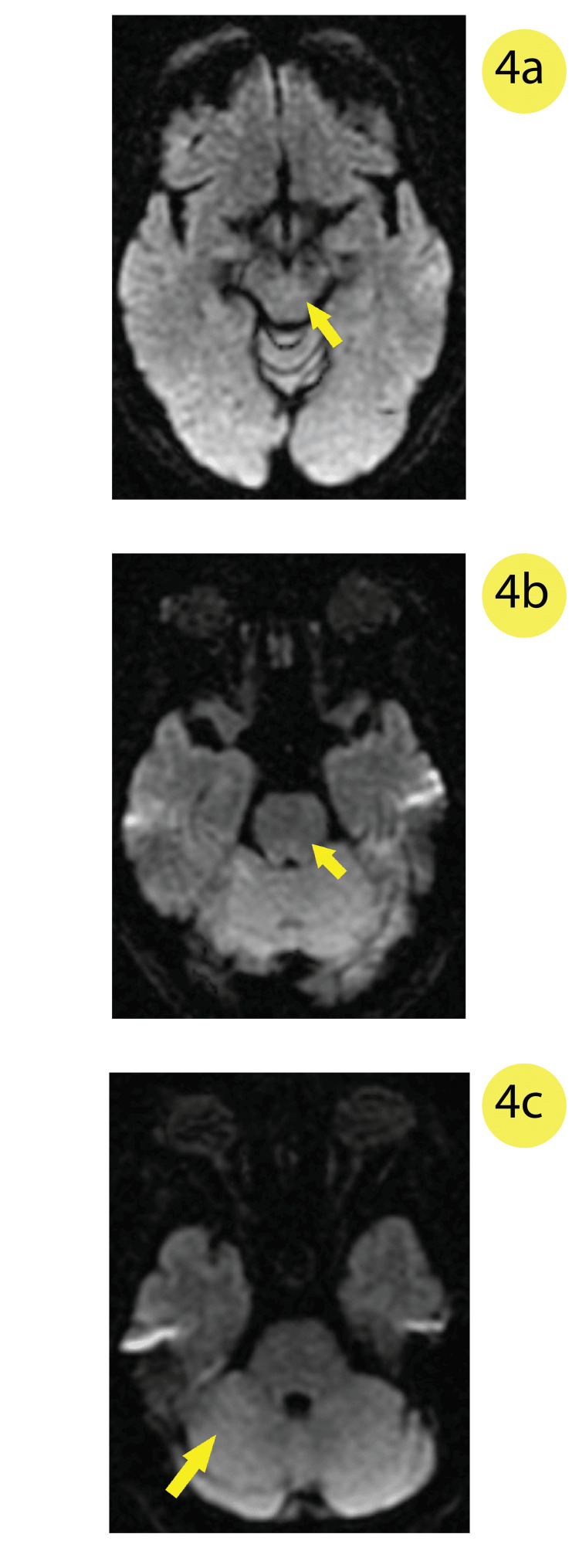
Diffusion-weighted image (DWI) MRI showing no diffusion restriction in the a) central midbrain, b) central-posterior pons, or c) the right superior cerebellum

The patient’s bilateral posterior communicating arteries were present, supplying the top of the basilar artery as well as the posterior cerebral arteries and the left-sided superior cerebellar artery. The initial CT head report suggested a questionable lesion in the left frontal lobe (Figure 3), but, because this was not present throughout all the CT images, MRI with and without contrast was ordered for further evaluation of the BAO and the possible left frontal lesion. The brain MRI was completed and showed no acute infarcts or ischemia (Figure 4). The possible contrast-enhancing lesion in the left frontal lobe was not seen on the MRI. After imaging was completed, the patient was admitted under the care of an inpatient hospital team for further management. Aspirin 81mg daily and high-intensity statin was started.

On the evening of admission, the patient developed an acute feeling of illness, worsening nausea and vomiting, vertigo, perioral and left-hand numbness, and light-headedness. The primary team called the neurology team, stating that she had worsening mental status and had become more somnolent. She was then transferred to the intensive care unit (ICU) for neuro checks every hour. A CT angiogram was ordered and showed stable findings compared to the morning. Her neurologic exam at this time was significant for disorientation where she was confused as to where she was, bradyphrenia, moderate-severe dysarthria with trouble remembering words and muffled speech, left-sided ptosis, and bilateral mild ataxia with finger-nose-finger. She was started on a heparin drip, as it was suspected that propagation of the BAO was the cause of her deterioration. She was nil per mouth for the next three to four hours. Our neurology team was in contact with the interventional radiology team, and, after a discussion with the patient and her family, it was decided that interventional radiology would perform a mechanical thrombectomy. The procedure was started within an hour of these new symptoms onset. The patient tolerated the procedure with no acute complications and was sent back to the ICU for monitoring. The heparin drip was restarted immediately after the procedure.

The next morning, the patient was almost back to her baseline. She had no deficits on neurologic examination but did report some mild dizziness. A repeat head CT to check on the occlusion and for any intracranial bleeds was negative. Interestingly, she had started to report vivid visual hallucinations of her daughter in a bathing suit at the beach, but she knew that the images she saw could not be real. That afternoon, a CT angiogram showed a widely patent basilar artery with no evidence of residual distal embolus. The heparin drip was discontinued approximately after 24 hours, and she was restarted on her home anti-coagulation regimen. She was eventually discharged back to the primary medicine inpatient service.

**Figure 5:**
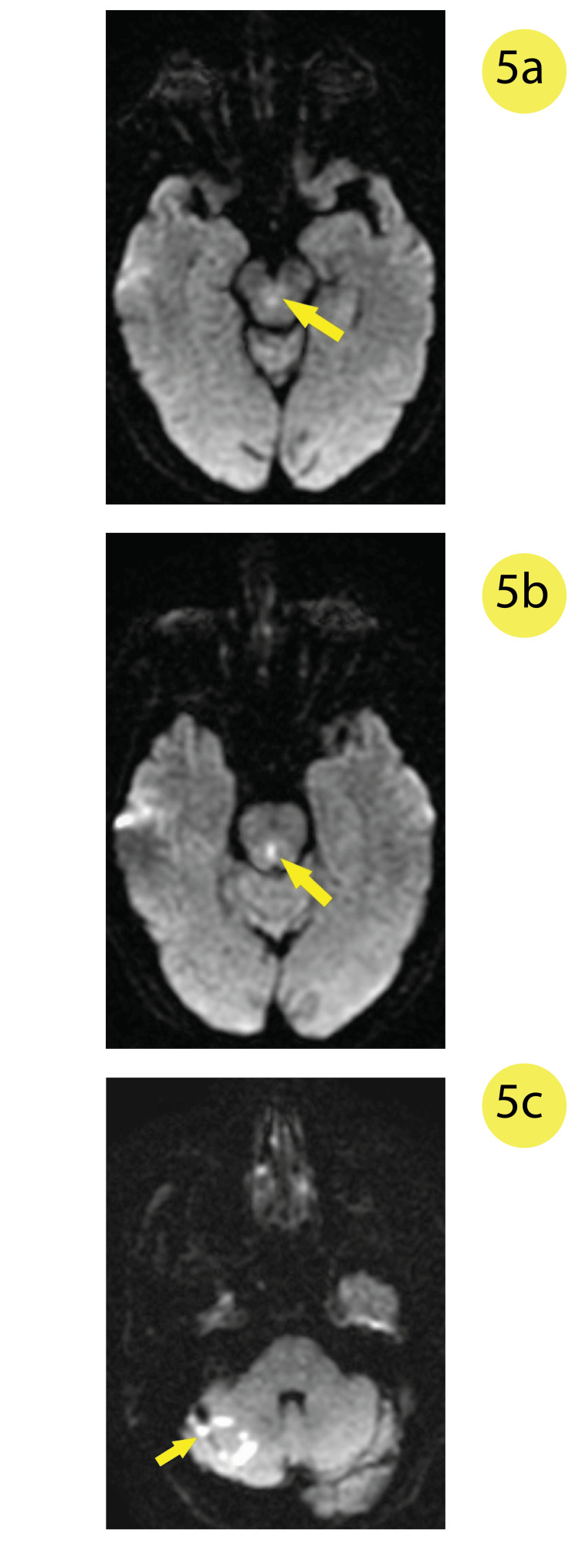
Diffusion-weighted image (DWI) MRI of the brain showing diffusion restriction in the a) central midbrain, b) central-posterior pons, and c) the right superior cerebellum

An MRI of the brain was performed on the morning of the patient’s third day at the hospital, which showed diffusion restriction in the bilateral cerebellar hemispheres and central midbrain (Figure 5) consistent with her deficits appreciated before she was transferred to the ICU. The questionable left frontal lobe lesion was not appreciated on this MRI scan. Her visual hallucinations and dizziness resolved, and the patient was eventually sufficiently stable for discharge a few days later.

On her outpatient clinic follow-up appointment six weeks after discharge, the patient’s condition improved significantly. Her neurologic examination was unremarkable, and no deficits were appreciated. Her speech was baseline. Finger-nose testing was normal on both sides. She denied any dizziness, numbness or tingling sensations, weakness, or visual hallucinations. She reported that her quality of life had been restored. Her long-term plan comprises stroke risk reduction with rivaroxaban and high-intensity statin, as well as blood pressure management.

## Discussion

“Time is the brain,” and time lost is brain lost - a statement that summarizes our case report, encompasses the main goal of BAO treatment, and highlights the importance of timely intervention to preserve brain tissue. Studies show that the benefit of endovascular therapy for large vessel occlusion decreases as the time to treatment increases, but at the same time, benefit of Endovascular therapy in delayed time windows has also been established [1]. These outcomes can also be modified based on patient’s collateral circulation and the salvageable Ischemic penumbra [7].

Our patient’s LKN was 22:30 the night before hospitalization, and interventional radiology performed mechanical thrombectomy approximately 23 hours later around 21:15 on the patient’s first day in the hospital. Our case report indicates that mechanical thrombectomy can still be beneficial for patients with distal BAO at 23 hours from their LKN, which corresponds with the prior literature [8,9]. Basilar artery strokes comprise 1% of posterior circulation strokes, however, the mortality rate is greater than 85% in patients without recanalization. The outcomes improve to 24-35% after endovascular interventions [10]. As noted by her outpatient clinic follow-up, the patient’s neurologic deficits had completely resolved, which may not have been the case had a mechanical thrombectomy not been performed. Mechanical thrombectomy is an endovascular technique to pursue direct removal of clots from the blood vessels after an Ischemic stroke. A catheter via groin is progressed to the cerebral blood vessels and pulls the clot out using a mesh device that entangles the clot. It can only be done in cases of large vessel occlusions. The contraindications to mechanical thrombectomy include hemorrhagic strokes, large infarcted core with no penumbra, strokes in small vessels, untreatable bleeding disorders and high blood pressure (>180 systolic, or >110 diastolic) that can not be corrected. Complications to this procedure dislodging of the clot distal to the block, stenosis of the vessel, vessel perforations, groin hematomas and recurrence of occlusion [10].

Our patient also displayed a curious neurologic phenomenon called “peduncular hallucinosis,” which is described as vivid visual hallucinations in an otherwise ordinary patient with normal cognitive function. Peduncular hallucinosis is typically due to a lesion in the brainstem (most commonly the midbrain) but can also result from a lesion in the thalamus [7]. It typically resolves over time, and, in the case of our patient, it improved after several days. Patient’s NIHSS was 1 in this case, however, it is important to note that NIHSS is mostly used to assess anterior circulation symptoms rather than posterior circulation [8]. Our patient’s anticoagulation was stopped one day before symptoms onset for her upcoming cataract surgery which might have contributed to her basilar artery occlusion. Per the literature, for ophthalmology-related surgeries, a meta-analysis showed an increased risk of bleeding (odds ratio=3.26; 95% confidence interval [CI], 1.73-6.16) in patients who continued the home chronic anticoagulation therapy that they were on. But it was shown that most of these bleeds were self-limiting with no compromise to the vision. This raises a question of whether the anticoagulation therapy should be continued in minor ophthalmological procedures [11]. A need to further our research on this topic is required, and discussing the risks and benefits of stopping anticoagulation prior to these procedures with the patients is of significance. 

This case report also highlights the importance of not relying too heavily on imaging. The patient had been symptomatic for approximately 10 hours before her first MRI of the brain, which did not show any findings (e.g., diffusion restriction on DWI or T2 fluid-attenuated inversion recovery [FLAIR] hyperintensities) to suggest that she was having an acute ischemic event as a result of the BAO. That evening, she developed acute symptoms consistent with brainstem ischemia, indicating that she had developed a brainstem stroke most likely due to the BAO. 

## Conclusions

Due to differences in vascular anatomy and chronic collateralization of blood flow around obstructions, BAOs can present with a wide range of symptoms. There must be a high index of suspicion on the initial exam, with imaging being confirmatory. However, the treating physician must be careful not to rely too heavily on imaging. Timely management of distal BAO is crucial to obtain the best overall prognosis.
